# Long-Term Memory for Pavlovian Fear Conditioning Requires Dopamine in the Nucleus Accumbens and Basolateral Amygdala

**DOI:** 10.1371/journal.pone.0012751

**Published:** 2010-09-15

**Authors:** Jonathan P. Fadok, Martin Darvas, Tavis M. K. Dickerson, Richard D. Palmiter

**Affiliations:** 1 Graduate Program in Neurobiology and Behavior, University of Washington, Seattle, Washington, United States of America; 2 Department of Biochemistry and Howard Hughes Medical Institute, University of Washington, Seattle, Washington, United States of America; Mental Health Research Institute and the University of Melbourne of Victoria, Australia

## Abstract

The neurotransmitter dopamine (DA) is essential for learning in a Pavlovian fear conditioning paradigm known as fear-potentiated startle (FPS). Mice lacking the ability to synthesize DA fail to learn the association between the conditioned stimulus and the fear-inducing footshock. Previously, we demonstrated that restoration of DA synthesis to neurons of the ventral tegmental area (VTA) was sufficient to restore FPS. Here, we used a target-selective viral restoration approach to determine which mesocorticolimbic brain regions receiving DA signaling from the VTA require DA for FPS. We demonstrate that restoration of DA synthesis to both the basolateral amygdala (BLA) and nucleus accumbens (NAc) is required for long-term memory of FPS. These data provide crucial insight into the dopamine-dependent circuitry involved in the formation of fear-related memory.

## Introduction

DA is synthesized by neurons in discrete nuclei within the brain, including the hypothalamus, olfactory bulb, and ventral midbrain [1]. DA neurons in the VTA of the ventral midbrain project to limbic brain areas that are important for fear conditioning, such as the prefrontal cortex, hippocampus, amygdala, and NAc [1], [2], [3]. Consistent with a role of DA in fear conditioning, the firing rate of DA neurons is altered by fear-inducing stimuli as well as cues that predict aversive outcomes [4], [5], [6]. Furthermore, in response to fearful stimuli or stressful situations, DA levels increase in several limbic brain regions [7], [8], [9], [10] and pharmacological and genetic manipulations of DA function can disrupt learning in fear conditioning paradigms [11], [12], [13], [14].

In Pavlovian fear conditioning, a neutral conditioned stimulus, such as a light, is paired with an aversive unconditioned stimulus, such as a footshock. Following training, presentation of the conditioned stimulus alone elicits fear responses [3]. FPS is a commonly employed Pavlovian fear conditioning paradigm in which learning is assessed by cue-elicited increases in the acoustic startle response [15]. We have previously demonstrated that DA neurons in the VTA are sufficient for learning in a FPS paradigm [12]. Furthermore, we demonstrated that DA in the BLA is sufficient to produce short-term memory (STM), but not long-term memory (LTM), of the cue-shock association. Of the remaining targets of VTA DA neurons, the NAc receives the largest innervation and was therefore a prime candidate site for the formation of LTM for FPS [2].

A large literature supports a role for DA within the NAc for associative learning processes in reward-based paradigms [16]. It is currently unclear whether DA in the NAc is also important for learning in Pavlovian fear conditioning. However, studies have shown that DA levels increase in the NAc in response to fearful stimuli and predictive cues [10]. Furthermore, the NAc is heavily innervated by the BLA [16], [17], a nucleus essential for fear conditioning, and DA facilitates neuronal function in both the NAc and BLA [18], [19], [20], [21]. Therefore, it is possible that connectivity between the BLA and NAc, and DA signaling in both of these regions, is required for Pavlovian fear conditioning.

To determine whether DA is necessary in the NAc and BLA for LTM in Pavlovian fear conditioning, we made use of the dopamine-deficient (DD) mouse model that lacks the ability to synthesize DA due to insertion of a loxP-flanked transcriptional/translational stop cassette in the *tyrosine hydroxylase (Th^fs^)* gene [22]. In the presence of Cre recombinase, DA signaling can be selectively restored to specific target regions by reactivation of the *Th^fs^* allele through the removal of the stop cassette. We used a retrogradely-trafficked virus expressing Cre recombinase to selectively restore DA to either the NAc alone, or to both the NAc and BLA. Our results demonstrate that DA in the NAc and BLA is sufficient for establishing LTM for FPS.

## Results

### Restoration of TH in Virally-Rescued DD Mice

To determine where in the brain DA is necessary for the formation of LTM for FPS, DA function was restored in DD mice via injections of CAV2-Cre recombinase. This virus selectively infects neurons and is retrogradely transported from the site of injection [23]. If injected into a target nucleus of DA neurons in DD mice, this virus will be trafficked back to DA neurons of the ventral midbrain where it excises the floxed stop cassette thereby reactivating the *Th* gene, restoring TH production, and allowing DA production only to the selected targets [22]. We used this technique in two separate cohorts of mice. Because the NAc is the largest target of DA neurons of the VTA [2], we hypothesized that this nucleus might be critical for the formation of LTM for FPS; therefore, bilateral injections of CAV2-Cre were made into the NAc in one cohort. We also tested the hypothesis that DA may be required in multiple targets of the VTA for LTM. To test this, bilateral injections were made into both the NAc and BLA of DD mice.

**Figure 1:**
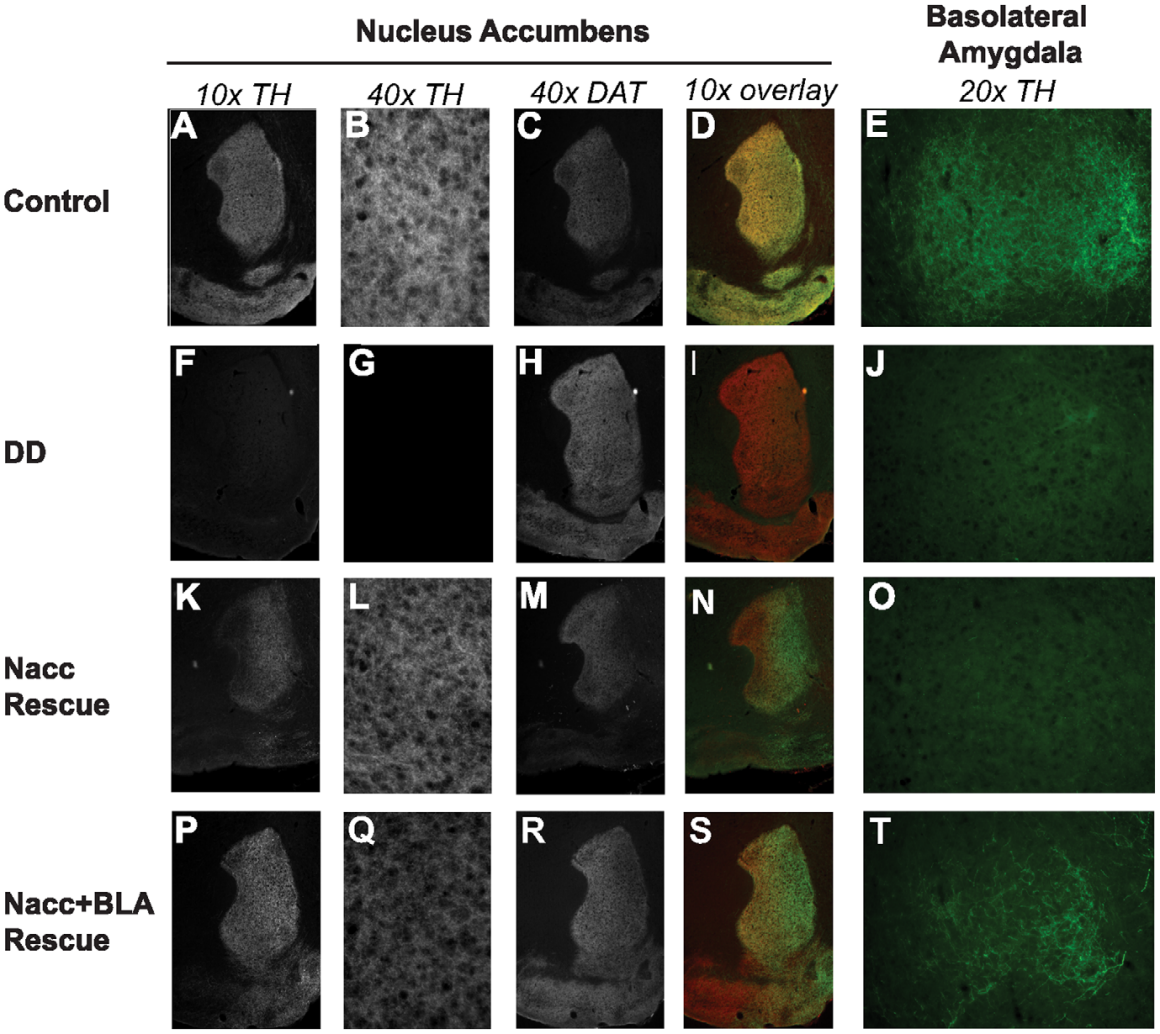
Selective restoration of TH in virally-rescued DD mice. *A–E)* Immunohistochemistry (IHC) results for a control mouse. *A)* Tyrosine hydroxylase (TH) stain in the nucleus accumbens (NAc). *B)* 40x magnification of TH stain. *C)* Stain for dopamine transporter (DAT) in NAc. *D)* Merged image of TH and DAT stain showing extensive overlap of the two signals. *E)* TH stain in basolateral amygdala (BLA) shown at 20x. *F–J)* IHC results for a non-rescued DD mouse. *F)* Complete absence of TH in NAc. *G)* 40x magnification demonstrating lack of TH in NAc. *H)* DAT stain. *I)* Merged image of TH and DAT illustrating lack of TH in the NAc. *J)* TH in BLA (20x) is almost undetectable. *K–O)* Representative IHC from NAc-rescued DD mouse. *K)* TH was largely restored in the NAc. *L)* 40x magnification illustrating rescue of TH. *M)* DAT staining in NAc-rescued DD mouse. *N)* Merged image of TH and DAT illustrating large extent of TH restoration in NAc. *O)* TH in BLA (20x) remains at low, non-injected levels. *P–T)* Representative IHC from DD mouse injected into the NAc and BLA. *P)* Robust restoration of TH to the NAc. *Q)* 40x magnification demonstrating TH rescue. *R)* DAT staining. *S)* Merged image of TH and DAT showing extensive restoration of TH to NAc. *T)* TH is restored to higher levels in NAc and BLA rescued DD mice.

Immunohistochemistry was used to confirm the restoration of TH function in virus-injected DD mice (Figure 1). As expected, there was a strong signal for TH in the NAc of control mice that co-localized with the DA transporter (DAT) (Figure 1A–D). TH was also detected in the BLA of control mice (Figure 1E); however, DAT immunoreactivity was very low in the BLA and is therefore not shown. Immunohistochemistry was also conducted on brain tissue from non-injected DD mice (Figure 1 F–J). There was no detectable TH signal in the NAc (Figure 1F, G), yet DAT staining was present (Figure 1H, I). The BLA of DD mice was also largely devoid of TH staining (Figure 1J).

Immunohistochemistry from NAc-injected DD mice showed that TH was restored to a large extent of the NAc (Figure 1K–N). No detectable TH was observed in the BLA of NAc-injected DD mice (Figure 1O). Double rescue to the NAc and BLA resulted in a robust signal for TH in the NAc (Figure 1P–S) and a strong TH signal in the BLA (Figure 1T). These data demonstrate that viral injection of CAV2-Cre was highly effective at restoration of TH expression specific to the brain regions injected.

To confirm that viral rescue of TH led to restoration of DA in injected DD mice, we quantified DA, DA metabolites and norepinephrine using high performance liquid chromatography (HPLC; Table 1). For this experiment, we performed rescue in either the NAc or the amygdala to also determine if TH rescue in one target of DA projections would influence DA levels in another, non-injected region. We found that dopamine-depleted DD mice had 0.51% of control DA levels in the NAc and 1.39% of control levels in the amygdala. NAc-rescued DD mice had DA levels that were 34.0% of control in the NAc; yet DA levels in the amygdala were the same as non-injected DD levels (1.57%). Amygdala-rescued DD mice had DA levels in the amygdala that were 38.4% of control, yet DA levels in the NAc were the same as non-rescued DD levels (0.46%). These results demonstrate that virus-mediated rescue of TH leads to elevated DA levels in injected target regions of DD mice. Furthermore, injection of virus into either the NAc or amygdala did not lead to elevation of DA levels in the other target. Finally, because TH is expressed in noradrenergic neurons of DD mice [24], [25], we attributed the small amount of TH seen in IHC of the BLA in DD mice to noradrenergic axons. The presence of norepinephrine in the BLA of non-rescued DD mice was confirmed with HPLC (Table 1).

**Table 1:** HPLC Quantification of DA, NE, and DA metabolites.

Group	*n*	Region	DA	DA	HVA	DOPAC	3-MT	NE
			(%Control)	(ng/mg)	(ng/mg)	(ng/mg)	(ng/mg)	(ng/mg)
Control	3	NAc	100	71.5±4.64	6.13±0.67	10.3±2.09	7.47±2.50	0.34±0.22
Control	3	AMYG	100	16.0±6.44	2.56±0.62	2.29±0.5	2.79±0.63	0.95±0.46
DD	4	NAc	0.51	0.37±0.15	0.84±0.40	0.63±0.62	1.17±0.26	0.94±0.27
DD	4	AMYG	1.39	0.22±0.03	0.62±0.06	0.08±0.04	1.99±1.10	2.19±1.21
NAc-Rescue	4	NAc	34.0	24.3±3.01	1.7±0.21	3.17±0.63	2.50±0.52	0.47±0.32
NAc-Rescue	4	AMYG	1.57	0.25±0.02	0.33±0.10	0.10±0.04	1.64±0.39	1.43±0.15
AMYG-Rescue	4	NAc	0.46	0.33±0.08	1.68±1.35	1.24±1.23	2.28±0.11	0.69±0.32
AMYG-Rescue	4	AMYG	38.4	6.12±3.19	1.48±0.52	0.68±0.23	2.21±0.87	1.79±0.25

DA, dopamine; HVA, homovanillic acid; DOPAC, 3.4-dihydroxyphenylacetic acid; 3-MT, 3-methoxytyramine. Data presented as means ± SEM.

### Dopamine is Required in the NAc and BLA for Long-Term Memory

**Figure 2:**
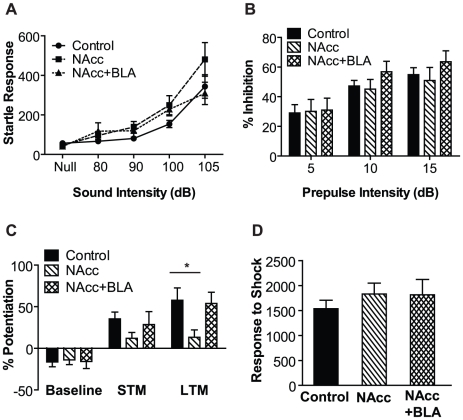
Restoration of DA to both the NAc and BLA is sufficient for LTM for FPS. *A)* Startle response curves for all three groups of mice (control, n = 24; NAc alone, n = 13; Nacc+BLA, n = 9) illustrating intact startle responses in all rescue groups. *B)* Prepulse inhibition is intact in all groups of mice. *C)* LTM for fear-potentiated startle is restored to control levels in NAc and BLA-rescued DD mice but not in NAc-alone rescues. * = p<0.05, Bonferroni post-test. *D)* Behavioral response to footshock was the same in all groups.

Fear-potentiated startle is a form of Pavlovian conditioning in which a conditioned stimulus elicits increases in the acoustic startle response [15]. To ensure that selective restoration of DA only to the NAc, or only to the NAc and BLA, does not impair the acoustic startle response itself, startle response curves were generated for controls and rescued DD mice (Figure 2A). Two-way repeated measures analysis of variance (RM ANOVA) revealed a significant effect of sound intensity (F_(4,172)_ = 37.1, p<0.01), but no group by treatment interaction. Perturbations of DA function can also cause differences in sensorimotor gating that could impair FPS [15], [26]. To analyze sensorimotor gating, all mice were tested at multiple levels in a prepulse inhibition (PPI) paradigm (Figure 2B). There was a significant effect of prepulse intensity (RM ANOVA F_(2,86)_ = 57.79, p<0.01) but no group by treatment interaction. These results demonstrate that the selective rescue of DA signaling to the NAC, or NAc and BLA, caused by our experimental manipulations did not change the acoustic startle response or sensorimotor gating.

The mice were subjected to a fear conditioning paradigm (Figure 2C). During training, mice were given 30 trials in which a 10-sec light cue was paired with a mild footshock (0.5 sec, 0.2 mA). Short-term memory (STM) was tested 10 min after training and LTM was tested 24 hr later. There were no significant differences between groups before conditioning. Following training, STM was completely restored in DD mice with restoration to the NAc and BLA. STM in NAc-injected DD mice was impaired, yet this effect failed to reach significance; however, they had significantly less LTM than control mice (p<0.05; Bonferroni posttest). LTM was completely restored to control levels in DD mice injected bilaterally into both the NAc and BLA. There were no significant differences between groups in behavioral reaction to footshock (Figure 2D). These data demonstrate that DA in the NAc and BLA is sufficient to facilitate LTM for FPS.

## Discussion

DA is thought to facilitate consolidation and the formation of LTM in key limbic brain regions such as the amygdala, NAc and hippocampus [27], [28], [29], and previous studies have suggested a role for DA in Pavlovian fear conditioning [13]. Previously, we demonstrated that DA is critical for stabilizing the memory trace in a FPS paradigm [12]. Furthermore, restoration of DA function to the mesocorticolimbic circuit emanating from the VTA was sufficient to restore STM and LTM for FPS, yet restoration to the BLA alone only restored STM [12]. However, the sites of DA action required for formation of LTM in this type of learning was unknown. Here, we demonstrate that restoration of DA synthesis to the NAc and BLA is sufficient for LTM for FPS. We also find that restoration of TH to DA neurons projecting to the NAc was not as effective at rescuing STM as BLA restoration [12], or restoration to both the BLA and NAc. This suggests that the NAc might be more important for the formation of LTM than STM.

One potential caveat to the viral restoration approach is that DA neurons could send collateral projections to more than one target. Thus, injecting virus into the NAc could restore TH, and thereby DA, to the BLA. Our immunohistochemistry results suggest that the DA neurons innervating the NAc are a distinct population from those innervating the BLA because injecting the virus in one brain region enhanced TH staining only in that region. The HPLC results strengthen this argument because DA levels are elevated in the NAc of NAc-rescued DD mice and not in the amygdala. These findings are consistent with numerous studies that have explored the heterogeneity of DA neurons based on projection target [30], [31], [32], [33].

The circuitry and mechanisms underlying the need for DA in both the NAc and BLA for Pavlovian fear conditioning remain unresolved. Intriguingly, the BLA sends projections to the NAc [16], [34] and these synapses can undergo long-term potentiation, a key cellular correlate of learning and memory [35]. Moreover, DA facilitates LTP in the BLA and NAc [18], [21]. Thus, during Pavlovian fear conditioning, it is possible that DA in the BLA facilitates glutamatergic pyramidal cell activity [19], [20], [36], including those cells which project to the NAc [34], while DA in the NAc facilitates LTP of BLA to NAc synapses, thereby promoting the formation of LTM. Determining the precise timing of DA-dependent events in the BLA and NAc for FPS will enhance our understanding of this process.

## Materials and Methods

### Ethics Statement

All mice were treated in accordance with guidelines established by the National Institutes of Health and procedures with mice were approved by the University of Washington Institutional Animal Care and Use Committee (2183-02).

### Animals and treatments

DD mice were generated as described [22]. Briefly, DD (*Th^fs^* ^/*fs*^; *Dbh^Th^* ^/+^) mice carry two inactivated tyrosine hydroxylase (*Th)* alleles which can be conditionally reactivated by Cre recombinase. DD mice have one intact dopamine β-hydroxylase (*Dbh*) allele, and one *Dbh* allele with targeted insertion of the *Th* gene to allow for normal production of norepinephrine [24], [25]. Control animals carry at least one intact *Th* allele and one intact *Dbh* allele. Male and female mice were subjected to behavioral testing between the ages of 2–6 months. All mice were housed under a 12∶12 (light:dark) cycle in a temperature-controlled environment with food (5LJ5; PMI Feeds, St. Louis, MO) and water available *ad libitum*. All behavioral experiments were conducted during the light cycle. Because DD mice are severely hypophagic, they were injected daily (intraperitoneally)with 3, 4-dihydroxy-L-phenylalanine (L-Dopa)at 50 mg/kg at a volume of 33 µl/g, starting at approximately post-natal day 10 [25]. After viral injection, DD mice were maintained with daily injections of L-Dopa until they could eat adequately without further L-Dopa treatment.

### Viral Injections

Isoflurane (1.5–5%)-anesthetized mice were placed into a stereotaxic instrument (David Kopf Instruments, Tujunga, CA). For restoration of *Th* gene function in the nucleus accumbens alone, recombinant *CAV2-Cre* virus (titered at 2.1×10^12^ particles/ml) was injected bilaterally (coordinates in mm: 1.7 anterior to Bregma, 0.75 lateral to midline, 4.75 ventral to Bregma; 0.5 µl/hemisphere) into DD and control mice. For double restoration of DA to the NAc and BLA, CAV2-Cre virus was injected bilaterally into the NAc, as above, and the BLA (coordinates in mm: 1.5 posterior to Bregma, 3.25 lateral to midline, 5 ventral to Bregma; 0.5 µl/hemisphere) in DD and control mice. Detailed description of this viral vector has been published [22]. Viruses were injected over a 10-min period using a 32-gauge syringe needle (Hamilton, Reno, NV) attached to a micro-infusion pump (WPI, Sarasota, FL). Control mice from NAc alone and double rescue cohorts were compiled into one group and did not differ in any behavioral parameter.

### Apparatus

Sound-attenuating startle chambers (SR-Lab, San Diego Instruments, San Diego, CA) were used to measure prepulse inhibition, startle responses, and fear-potentiated startle, as described [12]. The peak amplitude of the response was used to calculate prepulse inhibition, startle responses, fear-potentiated startle, and shock reactivity. Sound levels were verified using a sound level reader (RadioShack, Fort Worth, TX). A calibration unit was used to ensure the integrity of the startle response readings (San Diego Instruments, San Diego, CA). An 8-watt light was mounted on the rear wall of the startle box for use as a cue.

### Startle response curves

Following a 5-min habituation period, animals were presented with 10 series of trials with escalating sound pulse levels: from null, in which there was no sound, to 105 dB, with an ITI of 30 sec. All sound pulses were 40 msec.

### Pre-pulse inhibition

PPI was measured as described [12]. Briefly, following a habituation period, mice were presented with 5, 40-msec, 120-dB, pulse-alone trials. Mice were then presented with 50 trials of either a startle pulse-alone trial, one of three prepulse trials (5, 10, and 15-dB above background), or a null trial in which there was no acoustic stimulus. Prepulse inhibition was calculated for each prepulse level using the following formula: % inhibition = [(average startle response on prepulse trial/average startle response on pulse-alone trial) ×100].

### Fear-potentiated startle

All mice were tested using the 3-day FPS paradigm as described [12]. Briefly, on baseline, mice were given a pseudo-randomly ordered series of 20 trials, split evenly between cue and no-cue conditions. On day 2, mice received 30 pairings (2 min mean ITI) of the 10-sec cue light with a 0.2-mA, 0.5-sec footshock. The mice were then placed into their home cages for 10 min before testing for short-term memory. On day 3, LTM was assessed. The following formula was used to calculate fear-potentiated startle: %potentiation = [(average of responses on cue trials/average of responses on no-cue trials-1) ×100].

### Immunohistochemistry

Mouse brain tissue was prepared for histological analysis using standard techniques, as described [12]. Free-floating coronal sections (30 µm) were immunostained with rabbit anti-TH (1∶2000, Millipore) and rat anti-DAT (1∶1000, Millipore) antibodies. Secondary antibodies were either Cy2- or Cy3- conjugated (1∶200, Jackson ImmunoResearch). Photographs were taken with an upright brightfield microscope (Nikon).

### High-performance liquid chromatography

Mice were euthanized with Beuthanasia (250 mg/kg) and then brains were removed and placed on an ice-cold marble plate. Using a mouse brain matrix (Activational Systems, Warrren, MI), 1-mm thick slices were taken through the NAc or amygdala. Tissue punches (1-mm diameter) were then taken, placed into 1.7 mL microcentrifuge tubes, and quickly frozen in liquid nitrogen. Samples were stored at −80°C until they were shipped on Dry Ice to Neurochemistry Core Lab (Venderbilt University Center for Molecular Neuroscience Research) for analysis.

### Statistical Analyses

Statistical analysis was performed using GraphPad Prism software (La Jolla, California).
